# Mucosal Immune Development in Early Life: Setting the Stage

**DOI:** 10.1007/s00005-015-0329-y

**Published:** 2015-02-11

**Authors:** Sylvia Brugman, Olaf Perdijk, R. J. Joost van Neerven, Huub F. J. Savelkoul

**Affiliations:** 1 Cell Biology and Immunology Group, Wageningen University, de Elst 1, 6708 WD Wageningen, The Netherlands; 2 FrieslandCampina, Amersfoort, The Netherlands

**Keywords:** Mucosal immunity, Development, Airways, Fetal, Neonatal feeding, Inflammatory bowel disease

## Abstract

Our environment poses a constant threat to our health. To survive, all organisms must be able to discriminate between good (food ingredients and microbes that help digest our food) and bad (pathogenic microbes, viruses and toxins). In vertebrates, discrimination between beneficial and harmful antigens mainly occurs at the mucosal surfaces of the respiratory, digestive, urinary and genital tract. Here, an extensive network of cells and organs form the basis of what we have come to know as the mucosal immune system. The mucosal immune system is composed of a single epithelial cell layer protected by a mucus layer. Different immune cells monitor the baso-lateral side of the epithelial cells and dispersed secondary lymphoid organs, such as Peyer’s patches and isolated lymphoid follicles are equipped with immune cells able to mount appropriate and specific responses. This review will focus on the current knowledge on host, dietary and bacterial-derived factors that shape the mucosal immune system before and after birth. We will discuss current knowledge on fetal immunity (both responsiveness and lymphoid organ development) as well as the impact of diet and microbial colonization on neonatal immunity and disease susceptibility. Lastly, inflammatory bowel disease will be discussed as an example of how the composition of the microbiota might predispose to disease later in life. A fundamental understanding of the mechanisms involved in mucosal immune development and tolerance will aid nutritional intervention strategies to improve health in neonatal and adult life.

## Fetal Life

### Sterile or Not?

Previously it was thought that the fetal environment in the uterus was sterile and the fetal immune system was immature and inactive. However, in recent years, more and more evidence has emerged that the fetus is actually exposed to environmental antigens prior to birth and that the contact between the immune system of mother and child is far more intimate than previously thought. Here, we will summarize the most recent data (see also Table 1).

**Table 1:** Environmental factors influencing host immunity during fetal and neonatal life

Factor	Specific substance	Immunological mechanism/clinical effect on host	Model	References
Fetal life				
Placental microbiota		APCs epigenetically regulate RORγt expression in umbilical cord T cells	Human	(de Roock et al. 2013; Stoppelenburg et al. 2014)
	Microbial-derived riboflavins	Fetal intestinal MAIT cells produce IFN and IL-22	Human	(Corbett et al. 2014; Kjer-Nielsen et al. 2012; Le Bourhis et al. 2010; Leeansyah et al. 2014; Treiner et al. 2003)
Amniotic fluid	AMPs	Bacterial lytic effects	Human	(Cherry et al. 1973; Espinoza et al. 2002; Kim et al. 2002)
	Endotoxin-neutralizing AMPs	Preventing TLR signaling	Human	(Kim et al. 2002)
	EGF	Preventing TLR signaling	Human	(Good et al. 2012)
Maternal factors	Cells that cross the placenta	Induction Tregs in secondary lymphoid tissue	Human	(Mold et al. 2008)
	Consumed vegetables	Less intraepithelial lymphocytes and RORγt^+^ ILCs	Mice	(Kiss et al. 2011; Lee et al. 2012; Li et al. 2011)
	Probiotics (*B. lactis* and/or *L. rhamnosus* GG)	Altered TLR expression in exfoliated cells	Human	(Rautava et al. 2012)
*Microbial colonization	(Table 2)			
Neonatal life				
Breast milk	Growth factors	Increased epithelial barrier functioning	Human	(Wagner et al. 2008)
	Lactoferrin	Anti-microbial	Human	(de Oliveira et al. 2001; Giugliano et al. 1995)
	Oligosaccharides	Improve diversity and microbial metabolism	Human/mice	(Oozeer et al. 2013; Scholz-Ahrens et al. 2007; Scholz-Ahrens and Schrezenmeir 2007)
	Milk glycans	Protection from enteric pathogens	Human/mice	(Newburg 2005, 2012)
	Insulin-like growth factors	Wound healing and tissue repair	Rats	(Clark et al. 2006; Halpern et al. 2003)
	Epidermal growth factors	Anti-inflammatory and induced mucus production	Rats	(Clark et al. 2006; Halpern et al. 2003)
	Commensal bacteria	Inhibition pathogens?	Human	(Heikkila and Saris 2003; Hunt et al. 2011; Martin et al. 2009)
	IgA	Humoral immunity/modulates microbiota composition	Human	(Rogier et al. 2014; Rogosch et al. 2012; Wolf, et al. 1994, 1996)
Raw cow milk/	bIgG	Recognizes pathogens that can also infect humans (e.g. RSV)	Human	(den Hartog et al. 2014)
Collostrum	bIgG	Reduces recurrent diarrhea in AIDS patients	Human	(Floren et al. 2006)
	Lactoferrin, lactoperoxidase and lysozyme	Protects low birth weight infants from necrotizing enterocolitis	Human	(Manzoni et al. 2014)
Vitamin A		Establishes normal levels of type 3 (RORγT^+^) intestinal lymphoid cells	Mice	(Spencer et al. 2014)
	Retinoic acid (+ TGF-β)	Promotion of Tregs via CD103^+^ DCs	Human/mice	(Coombes et al. 2007; den Hartog et al. 2013)
	Retinoic acid	Inhibits Th17/converts Tregs to T follicular helper cells/upreg. CCR9 and α4β7	Mice	(Benson et al. 2007; Iwata et al. 2004; Mora et al. 2003; Mucida et al. 2007; Sun et al. 2007; Takahashi et al. 2012)
	Retinoic acid	Induce IgA-secreting B cells	Human/mice	(Mora et al. 2006)
	miR-10a induced by retinoic acid	T-bet expression/Th1 immunity	Mice	(Takahashi et al. 2012)
Vitamin D		Increase CD8αα^+^ intraepithelial T cells	Human	(Kang et al. 2012)
		Treg induction by binding of VDR-RXR to enhancer of Foxp3 gene	Mice	(Bruce and Cantorna 2011)
Fermentation products	SCFAs	Recruitment of leukocytes and T cell activation	Mice	(Brown et al. 2003; Kim et al. 2013)
Starch	Butyrate and acetate	Treg differentiation via colonic DCs and macrophages (via GPR109A receptor)	Mice	(Singh et al. 2014)
	Butyrate	Anti-inflammatory: epigenetically (HDAC, FOXp3)/reduced chemotaxis of monocytes	Human/mice	(Han et al. 2007; Meijer et al. 2010; Park et al. 2004; Quivy and Van Lint 2004)
	Acetate or propionate	Reduce LPS-induced TNF release from neutrophils	Human/mice	(Tedelind et al. 2007)
Vegetables	Glucosinolates (e.g. TCDD)	Epigenetic modulation of Foxp3 and RORγT^+^ genes (via aryl hydrocarbon receptor)	Mice/rats	(Bjeldanes et al. 1991; Singh et al. 2011)

For example, bacteria belonging to the genus of *Enterococcus, Streptococcus, Staphylococcus*, and *Propionibacterium* could be cultured from umbilical cord blood of healthy neonates born by cesarian section (Jimenez et al. 2005). Additionally, while cultivation of the placental samples did not reveal the presence of viable bacteria, *Bifidobacterium* and *Lactobacillus* DNA could be detected in 33 and 31 of 34 placenta samples, respectively (Satokari et al. 2009). In a recent study, 320 placental samples were analyzed by comparative 16S ribosomal DNA-based and whole-genome shotgun metagenomics. Here, the authors report that the placenta harbors a unique microbiome consisting of several non-pathogenic bacteria. This placental microbiome mostly resembled the mother’s oral microbiome (Aagaard et al. 2014). The placenta, therefore, might harbor several antigens to which the fetus needs to develop tolerance (Zaura et al. 2014). Furthermore, lactic acid bacteria and enteric bacteria have been found in the meconium, the first fecal discharge of neonates that was thought to be sterile (Jimenez et al. 2008). These data suggest that bacteria or at least bacterial DNA can come in contact with fetal tissues and this does not automatically lead to premature birth or spontaneous abortion. Thus, during fetal life overt inflammatory responses towards environmental or maternal (commensal) bacteria must be prevented, to forestall premature birth or death of the fetus.

### Underdeveloped or Repressed Immunity?

Stoppelenburg et al. (2014) have shown that umbilical cord blood T cells fail to differentiate toward the pro-inflammatory Th17 lineage in the presence of autologous antigen-presenting cells. In a separate study, they also showed that neonatal T cells have an intrinsic mechanism that prevents Th17 differentiation through the regulation of RORγt expression, possible via DNA methylation and histone acetylation (de Roock et al. 2013). This again indicates that overt inflammatory responses are actively repressed in the fetus and neonate. At the same time, this might pose a risk to mother and child. Indeed, it has been shown that pregnant women have a 20-fold increased risk of developing listeriosis; infection with Listeria bacteria that causes infections of the central nervous system of the unborn, such as meningitis (Southwick and Purich 1996). This is probably due to repressed Th1 cell proliferation and interferon (IFN)-γ production during pregnancy (Southwick and Purich 1996).

To further prevent pro-inflammatory responses, the fetus is surrounded by amniotic fluid. This amniotic fluid contains anti-microbial peptides such as defensins and lactoferrin. Furthermore, it contains endotoxin-neutralizing histones and lipopolysaccharide (LPS)-binding protein that might prevent Toll-like receptor (TLR) signaling and possibly fatal immune responses for the unborn child (Cherry et al. 1973; Espinoza et al. 2002; Kim et al. 2002). Recently, it was shown in mice that epidermal growth factor (EGF) in the amniotic fluid inhibits fetal TLR signaling through binding to the EGF receptor on fetal intestinal epithelial cells (Good et al. 2012). So, instead of being underdeveloped and unresponsive, the fetus can respond to antigens, however, these responses are actively prevented.

### Development of Mucosal Lymphoid Tissue During Fetal Life

**Fig. 1:**
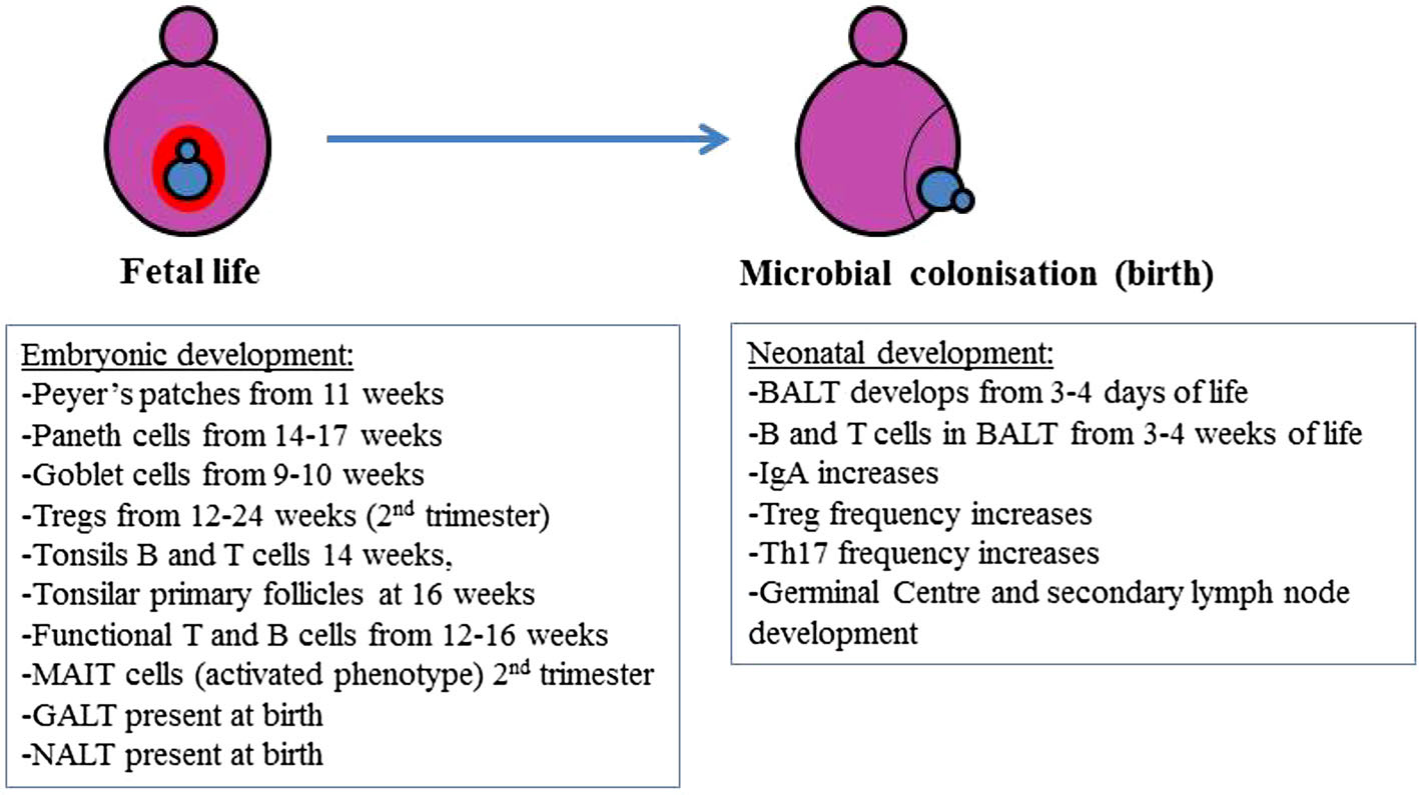
Development of mucosal immunity before and after birth. Contrary to what was believed, the fetal immune system contains mature T and B cells that are actively repressed by regulatory T cells. Of note, the gut-associated lymphoid tissue (GALT) and the nasal-associated lymphoid tissue (NALT) are present before birth, while the bronchial-associated lymphoid tissue (BALT) develops after birth

Meanwhile in the gut of the fetus, interspersed Peyer’s patches develop around 11 weeks of gestation and functional B and T cells can be found from 12 to 16 weeks, respectively (Fig. 1) (Cupedo et al. 2005; Darrasse-Jeze et al. 2005; Haynes et al. 1988; Hayward and Ezer 1974; Michaelsson et al. 2006; Mold et al. 2008). Both the gut-associated lymphoid tissue (GALT) and the intestinal epithelium mature during the gestational period. Specialized epithelial cells called Paneth cells develop in the colon and small intestine at 13.5 weeks of gestation. After 17 weeks, Paneth cells are confined to the small intestine (Poulsen et al. 1996). Paneth cells reside at the bottom of the crypts, secrete anti-microbial peptides and are important in protecting the intestinal stem cells and maintaining intestinal homeostasis (Bevins and Salzman 2011; Nieuwenhuis et al. 2009; Salzman et al. 2010). In the human fetal intestine, goblet cells appear around 9–10 weeks of gestation (Kim and Ho 2010). Goblet cells produce mucins that serve as a first line of defense against luminal antigens. In addition to mucins (large glycoproteins), mucus consists of water, ions and immune mediators such as immunoglobulin A (IgA) and anti-microbial peptides, which help clear pathogens (Hasnain et al. 2013; Phalipon et al. 2002). Early during development lymphoid precursor cells are present and spread to Peyer’s patches and mesenteric lymph nodes (Husband and Gleeson 1996). Memory T cells were found to be relatively abundant in fetal spleen and in cord blood samples from premature births. These cells comprised about 25 and 10 % of the T cells, respectively, expressed CD25 and were anergic (Byrne et al. 1994). At that time, 15–20 % of CD4^+^ T cells in the fetus’ secondary lymphoid tissues are comprised of Tregs. Murine studies suggest that these Tregs are largely induced by maternal cells that cross the placenta and reside in fetal lymph nodes (Mold et al. 2008). In this way, regulation of fetal anti-maternal immunity is established. The authors also suggest that this form of in utero-induced antigen-specific tolerance might also be active in regulating immune responses after birth (Mold et al. 2008). Next to the GALT, the nasopharynx-associated lymphoid tissue (NALT), and bronchus-associated lymphoid tissue (BALT) are also part of the mucosal-associated lymphoid tissue. The NALT (named Waldeyer’s ring in humans), consists of the nasopharyngeal tonsil, tubal tonsils, palatine tonsils and lingual tonsils (Perry and Whyte 1998). Its appearance is similar to Peyer’s patches; follicles underneath follicle-associated epithelium containing interspersed microfold cells that can sample antigens (Breel et al. 1988a, b). Tonsils are secondary lymphoid organs. The tonsillar subepithelial space is formed by several lymphoid follicles containing B and T cell areas. Tonsils are not encapsulated like the spleen, but are lined by tonsillar epithelium that invaginates forming crypts (Perry and Whyte 1998). From the 14th week of gestation, B and T cells populate the area under the tonsillar epithelium and primary follicles develop from 16 weeks of gestation (earlier than any other secondary lymphoid tissue). The tonsils will keep growing until 7 years of age after which they slowly involute (Passali 1992). While NALT is present at birth, BALT develops from 3 to 4 days of age (Breel et al. 1988a; Hameleers et al. 1989; Pabst and Gehrke 1990). It is not until 3–4 weeks of age until B and T cell areas are formed in the BALT (Breel et al. 1988a; Pabst and Gehrke 1990).

### Immune Modulation via Dietary or Bacterial Factors During Fetal Life?

Recently, a specific subset of T cells with an invariant receptor (mucosa-associated invariant T cells: MAIT) was also found to be present in the second trimester of human fetal tissues (Leeansyah et al. 2014). MAIT cells are innate-like T cells that recognize antigens in complex with the MHCIb-like protein MR1 (Treiner et al. 2003). MAIT cells recognize microbial-derived riboflavin metabolites and can subsequently produce IFN-γ, tumor necrosis factor (TNF) and interleukin (IL)-17 (Corbett et al. 2014; Kjer-Nielsen et al. 2012; Le Bourhis et al. 2010). Interestingly, these cells are present at high frequency in fetal lung, liver and small intestine, and display a mature phenotype (i.e., they express IL-18Rα^+^ and CD8αα) (Leeansyah et al. 2014). Compared to adult MAIT cells fetal small intestinal MAIT cells have an increased proliferative capacity and can respond to bacterial stimulation with production of IFN-γ and IL-22 (Leeansyah et al. 2014). The factors that drive this fetal MAIT maturation are currently unknown, but also might suggest that the human fetal environment is not devoid of external or environmental stimuli.

The fact that environmental factors can reach the fetal immune system via the placenta, suggests that fetal immunity might be altered or enhanced by dietary or microbial intervention in pregnant women. However, scientific evidence on the effect of dietary intervention in pregnant women on fetal immunity is limited. Rautava et al. (2012) report that women that received either *Bifidobacterium lactis* or *Bifidobacterium lactis* together with *Lactobacillus rhamnosus* GG 14 weeks prior to elective cesarean section showed altered TLR expression in the exfoliated cells present in the meconium of the newborn as compared to the placebo group. However, others have shown that dietary supplementation with probiotics during late pregnancy might alter maternal immune parameters, but does not alter fetal immune responses (Boyle et al. 2008; Vitali et al. 2012). Additionally, while supplementation with galacto-oligosaccharides and long-chain fructo-oligosaccharides alters maternal fecal microbiota (increase of bifidobacteria), it did not affect fetal immunity as measured by cord blood cell stimulation assays (Shadid et al. 2007). However, experiments performed with pregnant mice suggest that live bacteria can transfer from the mother to the fetus. Labeled *Enterococcus faecium* that were orally given to pregnant mice could be cultured from the amniotic fluid as well as from the mammary glands of the mothers (Jimenez et al. 2005). Interestingly, in mice treated with a diet devoid of vegetable material, decreased numbers of intraepithelial lymphocytes are seen as well as a reduction in type three innate lymphoid cells (RORγt^+^ ILC) in the intestines (Kiss et al. 2011; Lee et al. 2012; Li et al. 2011). Additionally, in a recent paper, van de Pavert et al. (2014) have shown in mice that maternal diet derived vitamin A induces lymph nodes in the unborn pups. Pups derived from mice fed vitamin A-deficient diets had markedly reduced lymph node size and decreased efficiency of immune responses. In this paper, van der Pavert showed that retinoic acid (the metabolite of vitamin A) is necessary for differentiation of lymphotoxin inducer cells that play a crucial role in lymph node formation (van de Pavert et al. 2014).

In conclusion, while increasing evidence suggests a direct interaction between the maternally derived environmental factors (such as diet and microbes) and the fetus, more research is warranted to investigate the mechanisms by which these factors might (beneficially) alter fetal and subsequent neonatal immunity.

## Neonatal Life

### Cesarean Section Versus Vaginal Birth: Effect on the Microbial Composition

During birth, the amniotic membranes rupture and the unborn child will passage through the birth canal. This birth canal is not sterile and during labor the child will get exposed to vaginal bacteria, maternal skin and feces followed by exposure to environmental antigens (Fanaro et al. 2003). This exposure has a profound impact on the host. Here, we summarize what is known in this interesting research field (see also Table 2).

**Table 2:** Effect of microbial colonization on host immunity

Factor	Microbial composition	Immunological mechanism/clinical effect on host	Model	References
Birth				
Vaginal birth	More *Bifidobacteria* and *Bacteroides*	Stronger humoral response (higher levels of IgA, IgG- and IgM-secreting B cells)	Human	(Biasucci et al. 2010; Huurre et al. 2008)
		Higher serum levels of sIL-2r and TNF	Human	(Malamitsi-Puchner et al. 2005)
Cesarean section	More *Klebiella*, *Enterobacter* and *Clostridia*	Higher risk of allergies (excl. inhalant atopy and eczema)	Human	(Adlerberth et al. 2006, 2007; Bager et al. 2008; Penders et al. 2006, 2007, 2013)
Bottle feeding	More intestinal *Bacteriodes* and *Clostridia*	Might predispose to development of autoimmunity, and childhood infections, atopy and asthma	Human	(Fallani et al. 2010; Fanaro et al. 2003)
	Oral microbiome without *Lactobacillus*		Human	(Holgerson et al. 2013, Vestman et al. 2013)
Breast feeding	More intestinal *Bifidobacteria*	Associated with protection from autoimmune disease, and childhood infections, atopy and asthma	Human	(Fallani et al. 2010; Fanaro et al. 2003; Vos et al. 2007)
	Oral microbiome with *Lactobacillus*		Human	(Holgerson et al. 2013; Vestman et al. 2013)
	Segmented filamentous bacteria	IgA plasma cells are restored to normal levels	Mice	(Cebra 1999; Crabbe et al. 1968)
	Bacteria from conventional raised mice	Increased Foxp3 expression in colitis model	Mice	(Strauch et al. 2005)
	Autologous bacteria	Tolerance induction that protects against IBD	Mice	(Duchmann et al. 1995)
	Altered Schaedler flora	Treg induction	Mice	(Hapfelmeier et al. 2010; Macpherson et al. 2005; Macpherson and Uhr 2004)
	*Bacteriodes fragilis*	Treg induction in a polysaccharide A-TLR2 dependent manner	Mice	(Round and Mazmanian 2010)
	*Faecalibacterium prautznitzii*	Enhances anti-inflammatory responses	Mice	(Qiu et al. 2013; Sokol et al. 2008)
	Cluster IV, XIVa and XVIII of *Clostridia*	Induce Treg frequency and inducible T-cell co-stimulator	Mice	(Atarashi et al. 2013)
	Segmented filamentous bacteria	More Th17 cells in small intestinal lamina propria, less in colon	Mice	(Gaboriau-Routhiau et al. 2009; Ivanov et al. 2009)

Studies comparing children born vaginally or by cesarean section have shown differences in microbial community and immune responses. For example, a Venezuelan cohort showed that most vaginally delivered infants acquired a bacterial composition dominated by *Lactobacillus*, *Prevotella*, or *Sneathia*; species that are found in their mothers vaginal microbiota (Dominguez-Bello et al. 2010). In contrast, infants born by cesarean section displayed a bacterial community dominated by *Staphylococcus, Corynebacterium,* and *Propionibacterium,* typical skin bacteria (Dominguez-Bello et al. 2010). A Finnish study compared the microbiota and antibody production at 1 month after birth and showed that children delivered by cesarean section harbored fewer *Bifidobacteria* and were shown to mount a stronger humoral immune response (Huurre et al. 2008). The authors reported that during the first year of life, infants born vaginally displayed lower total IgA-, IgG- and IgM-secreting B cells in peripheral blood. The mode of delivery also has been reported to affect serum cytokine levels. Malamitsi-Puchner et al. (2005) reported that soluble IL-2 receptor, IL-1β and TNF-α were significantly higher in cases of vaginal delivery than in cases of elective cesarean section in neonates at day 1 (IL-1β, IL-2 Receptor and TNF-α) and day 4 (IL-2R, TNF-α) of life. These two studies might suggest that children born vaginally have lower humoral and higher cellular immunity in early life, compared to children born by cesarean section. However, more data will be necessary to support this hypothesis. Several studies report increased abundance of *Bifidobacteria* and *Bacteroides* in vaginal-delivered children compared to children born by cesarean section (Biasucci et al. 2010; Huurre et al. 2008). Additionally, analysis of bacterial colonization from birth to 12 months of age in a cohort of Swedish, Italian and British infants using culturing techniques showed that children delivered by cesarean section displayed more *Klebsiella, Enterobacter*, and *Clostridia*, including the pathobiont *Clostridium difficile* compared to vaginally delivered babies (Adlerberth et al. 2006, 2007; Penders et al. 2006). Interestingly, studies performed in Western countries revealed that children born by cesarean section take 6 months to a year to acquire the same levels of *Bacteroides, Bifidobacteria* and *Escherichia coli* colonization as vaginally born children display directly after birth (Adlerberth et al. 2006; Hall et al. 1990; Penders et al. 2006). In contrast, children born by cesarean section in the developing world catch up much quicker indicating that the environment is an important factor in colonization patterns after birth (Adlerberth et al. 1991).

### Cesarean Section Versus Vaginal Birth: Effect on Allergic Diseases

Thus, from these studies it seems that vaginally born children harbor bacterial species that have been considered beneficial (*Bifidobacteria*), while children born by cesarean section are more prone to harbor species that are associated with, but do not necessarily lead to, disease (*E. coli* and *Clostridia*). Indeed, colonization with *Clostridium difficile* has been associated with a higher risk of a diagnosis of atopic dermatitis (Penders et al. 2007, 2013). Several meta-analyses have shown that babies born by cesarean section are at higher risk to develop allergy, including food allergies. Interestingly, in a Norwegian birth cohort, it was shown that children of allergic mothers who were born by cesarean section had a sevenfold increased risk of developing food allergy to egg, fish or nuts (Eggesbo et al. 2003). This effect was not seen in children whose mothers were not allergic indicating that a predisposition exists that together with birth by cesarean section can lead to food allergy. Likewise, in a German cohort, babies with a family history of allergy and born by cesarean section also showed an increased risk of allergic sensitization to food allergens compared with babies at risk born vaginally (Laubereau et al. 2004). Finally, a large meta-analysis in which 26 studies on the effect of delivery by cesarean section on one or more allergies were described showed that cesarean section was associated food allergy, atopy, allergic rhinitis, asthma, and hospitalization for asthma. However, they found no association with inhalant atopy and eczema/atopic dermatitis (Bager et al. 2008). Since children born by cesarean section have an altered bacterial community, it is generally thought that this altered microbiota can lead to differences in mucosal immune tolerance which can predispose to the development of allergies (Maynard et al. 2012). Indeed in Dutch cohort, colonization by *Clostridium difficile* (associated with cesarean section) at an age of 1 month was associated with wheeze and eczema in the first 6 years of life and with asthma from age 6 (van Nimwegen et al. 2011). Although the associations exist, reports on the mechanisms how these changes early in life lead to disease are understandingly scarce. However, from animal studies, we do know that exposure to certain bacterial species has an important impact on host immunity. In the next section, we will discuss the current knowledge of microbial modulation of host immunity generated using animal models.

### How do Colonizing Microbes Influence Host Immunity?

In the last decades, it has become clear that the composition of the microbial community has profound influence on our health. Most of this knowledge derives from studies using gnotobiotic experimental animals. These studies show that colonization by different microbial species early in life has clear effects on the development of the intestinal mucosal immune system. Interestingly, host responses to microbial colonization are highly conserved between species. A study investigating zebrafish responses towards colonization revealed 59 responses that are conserved between mouse and zebrafish. These responses included pathways involved in epithelial proliferation, promotion of nutrient metabolism, and innate immune responses (Rawls et al. 2004). Several immune cells and mediators are influenced by the microbiota, for example, germ-free mice that are devoid of bacteria have almost no IgA-secreting plasma cells. Only upon colonization with specific subtypes of bacteria, IgA plasma cells are restored to levels seen in conventionally raised mice (Cebra 1999; Crabbe et al. 1968). IgA is the predominant antibody secreted by plasma cells in the mucosal tissues (Pabst et al. 2008). Low-affinity, poly-specific IgA is believed to prevent adhesion of commensal bacteria to epithelial cells, while high-affinity, mono-specific IgA neutralizes toxins and pathogens (Hapfelmeier et al. 2010; Macpherson et al. 2005; Macpherson and Uhr 2004).

Studies have also shown that germ-free animals have altered Treg frequency. In a transfer model of colitis, it was shown that co-transfer of CD4^+^CD62L^−^ cells into SCID mice prevented colitis induced by CD4^+^CD62L^+^ cells only when those cells were derived from conventionally raised mice. The CD4^+^CD62L^−^ cells from germ-free animals were not able to suppress the colitis. This associated with a low expression of regulatory T cell marker Foxp3 in this population form germ-free mice (Strauch et al. 2005). Already in 1995, Duchmann et al. (1995) reported that hypo-responsiveness exists towards the hosts’ autologous bacteria. Lamina propria mononuclear cells and peripheral blood mononuclear cells (PBMCs) did respond towards heterologous intestinal microbes. In patients with inflammatory bowel disease this tolerance towards autologous bacteria was lost (Duchmann et al. 1995). Together these studies clearly indicated that Tregs are directly or indirectly induced by the intestinal microbiota.

Using the altered Schaedler flora (ASF), a mixture of eight bacterial species including *Lactobacilli, Bacteroides, Eubacterium, Mucispirillum, Fusiform* and *Clostridial* species, Macpherson and colleagues demonstrated that ASF colonization of germ-free mice increased the inducible Treg frequency in the colonic lamina propria by twofold (Hapfelmeier et al. 2010; Macpherson et al. 2005; Macpherson and Uhr 2004). Likewise, it was shown that *Bacteroides fragilis* was able to induce Tregs upon colonization. Interestingly, when germ-free mice were given *B. fragilis* devoid of polysaccharide A (*B. fragilis ΔPSA*), Tregs were not induced (Round and Mazmanian 2010). Further experiments showed that polysaccharide A induction of Foxp3 on CD4^+^ T cells required TLR2 activation (Round and Mazmanian 2010). Likewise, *Faecalibacterium prautznitzii* has also been demonstrated to enhance anti-inflammatory responses (Qiu et al. 2013; Sokol et al. 2008). This indicates that bacteria and their cell wall components are important mediators of immune cell differentiation. Recently, Atarashi et al. (2013) inoculated mice with a healthy human fecal sample, and selected for mice enriched in Treg-inducing species. From these selected mice, they isolated 17 strains of bacteria that were able to enhance Treg frequency and induce IL-10 and inducible T cell co-stimulator (ICOS) upon inoculation into germ-free mice. Identification of these 17 strains revealed that these bacteria were members of the clusters IV, XIVa and XVIII of *Clostridia*, which lack prominent toxins and virulence factors (Atarashi et al. 2013).

More evidence for the bacterial specific effects on immune development was reported by Ivanov et al. (2009) who have shown that the ability to increase the number of Th17 cells in the small intestinal lamina propria associated with the presence of segmented filamentous bacteria in mice (Gaboriau-Routhiau et al. 2009). Th17 cells are T cells that produce IL-17A, IL-17F and IL-22 and have been shown to play a role in inflammatory responses and host defense against bacterial and fungal pathogens (Bettelli et al. 2007; McKenzie et al. 2006; Ouyang et al. 2008). Conversely, in the colon lamina propria, it was shown that germ-free mice harbor more Th17 cells than conventionally raised mice. Upon microbial colonization epithelial cells produce IL-25, which in turn inhibits (either directly or indirectly) the expression of IL-23 by antigen-presenting cells (Zaph et al. 2008). IL-23 is a cytokine that is described to be necessary for Th17 pool maintenance (Zhou and Littman 2009). Reduction of IL-23, therefore, results in decreased numbers of Th17 cells in the colon. Likewise, Corbett et al. (2014) reported that bacteria with an active vitamin B2 (riboflavin) pathway generate epitopes that (in conjunction with host metabolites) can be recognized by the MAIT cells via MR1. This finding again illustrates that colonization by (specific subsets of) bacteria can give rise to different mucosal immune environments.

Recently, much attention has been directed towards a newly discovered cell subset: innate lymphoid cells (ILCs). Three types of ILCs have been identified: T-bet^+^ ILCs (including NK cells, ILC1), GATA3^+^ ILCs (ILC2) and RORγt^+^ ILCs (ILC3) (Sonnenberg and Artis 2012). These ILCs are in close contact with the microbes since they reside in between the epithelial cells (Maloy and Powrie 2011; Sonnenberg et al. 2011; Spits and Cupedo 2012; Spits and Di Santo 2011; Veldhoen and Withers 2010). While Gata3^+^ and T-bet^+^ ILC development does not seem to depend on microbial colonization, this is not completely clear for the RORγt^+^ ILCs. Some studies show normal development, while other show reduced frequency of RORγt ILCs in germ-free mice (Reynders et al. 2011; Sanos et al. 2009; Satoh-Takayama et al. 2011; Sonnenberg and Artis 2012; Sonnenberg et al. 2011; Vonarbourg et al. 2010). RORγt ILCs express TLR2 and can therefore directly be activated by bacterial ligands (Crellin et al. 2010).

In conclusion, colonization is an important process during which the immune system develops to a certain set-point in each individual. Therefore, colonization by *Bifidobacteria* or *Bacteroides* species (vaginally delivered children), might result in a different immune cell-repertoire (for example, T cell subsets) and distribution than colonization by *E. coli* (Cesarean section), thereby leading to a different immunological set-point that may or may not predispose (in combination with host genetic susceptibility) towards certain diseases.

## Dietary Exposure and Host Immunity in Early Life

### Bottle Feeding Versus Breastfeeding

Next to bacteria, the newborn encounters several new environmental antigens of which most will be derived from the diet. Therefore, children that will be breastfed will be exposed to different dietary antigens than those that will be bottle-fed. Human breast milk contains immunoglobulins, cytokines, growth factors, lysozyme, lactoferrin and a complex mix of milk oligosaccharides (Chatterton et al. 2013; Kosaka et al. 2010; Wagner et al. 2008). Breast milk and colostrum contain large amounts of IgA, but also immune cells and cytokines, and soluble TLR2 that might help restrict innate immune activation by microbes (LeBouder et al. 2003; Verhasselt 2010). In addition, breast milk contains growth factors that fortify the neonates’ epithelial barrier (Wagner et al. 2008). Lactoferrin in the breast milk can bind free iron, needed for bacterial growth, thereby reducing bacterial load. In addition, lactoferrin can prevent pathogenic bacteria (such as ETEC) from adhering to the epithelial cell layer through binding of *E. coli* colonization factors (de Oliveira et al. 2001; Giugliano et al. 1995). However, in the continuing battle between host and pathogens, several pathogenic species developed mechanisms to counteract the action of lactoferrin either by using receptors that can acquire iron from lactoferrin (*Neisseria*) or secrete proteins that specifically bind lactoferrin thereby preventing its function (*Streptococcus pneumoniae*) (Hammerschmidt et al. 1999; Ling and Schryvers 2006; Senkovich et al. 2007).

The structure of breast milk oligosaccharides has been shown to be very diverse and depend on several factors including diet, lifestyle, and ethnicity (Thurl et al. 2010). Oligosaccharides can improve diversity and rate of metabolism of the microbiota (Oozeer et al. 2013; Scholz-Ahrens et al. 2007; Scholz-Ahrens and Schrezenmeir 2007). Also, breastfeeding has an impact on the composition of the microbiota. Breastfeeding is associated with high numbers of *Bifidobacteria* in the gastrointestinal tract of the newborns, whereas bottle feeding resulted in more intestinal *Bacteroides* and *Clostridia* (Coppa et al. 2004; Fallani et al. 2010; Vos et al. 2007). Recently, it was shown that *Lactobacilli* could be cultured from saliva in 27.8 % of exclusively and partially breast-fed infants, but not from formula-fed infants (Holgerson et al. 2013; Vestman et al. 2013), indicating that the oral microbiome is also influenced by infant feeding (Zaura et al. 2014). Furthermore, it has been shown that human milk glycans can protect infants from enteric pathogens (Newburg 2005, 2012). Insulin-like growth factor is important for wound healing and tissue repair and EGF plays a role in cell proliferation and differentiation, induces mucus production by intestinal Goblet cells and can suppress pro-inflammatory cytokines (Clark et al. 2006; Halpern et al. 2003). Interestingly, human milk also contains bacteria. Culture-dependent mechanisms have shown the presence of *Staphylococcus, Streptococcus* and *Bifidobacterium* species (Heikkila and Saris 2003; Martin et al. 2009). Subsequently, sequence analysis has identified the presence of DNA from nine different bacterial genera (Hunt et al. 2011). Interestingly, recently it was reported that house dust mite allergen, DerP1, is present in human breast milk. Subsequent testing of breast milk containing DerP1 in a mouse model revealed that instead of protecting these mice from allergic responses, they were sensitized (Macchiaverni et al. 2014). This suggests that not only neonates are exposed to dietary antigens early in life via breast milk, they are also exposed to respiratory allergens via breast milk, and this does not always lead to tolerance to the antigens but may well result in sensitization.

Maternal IgA is reflective of the environment of mother and child and therefore can protect the newborn against possible pathogens that he or she might encounter right after birth. Maturation of the IgA-producing plasma cells slowly develops after birth. While, IgA H chain transcripts are found in cord blood as early as 27 weeks of gestation, at 60 weeks of age, somatic mutation frequency of IgA H chain transcripts only reaches 25 % of the adult values, with little evidence of Ag-driven selection (Rogosch et al. 2012). Therefore, maternal IgA from the milk will equip the newborn with antigen-specific humoral immunity at the time the child itself does not have a fully developed repertoire. Interestingly, recently it was shown in mice that breast milk-derived IgA modulates the composition of the microbial community in the gastrointestinal tract (Rogier et al. 2014). Next to preventing bacterial infections, maternal IgA can also reduce the oxidative burst and represses TNF-α and IL-6 production by human monocytes (Wolf et al. 1994, 1996).

### Protection from Disease?

There is a long debate in the literature about the possible beneficial effect of (prolonged and/or exclusive) breastfeeding for children at risk for type 1 diabetes. Already in 1984, Borch-Johnson et al. (1984) reported an inverse correlation between breastfeeding and incidence rates of childhood type 1 diabetes. Several other studies confirmed this correlation (Mayer et al. 1988; Rosenbauer et al. 2008), while others did not (Couper et al. 1999; Hummel et al. 2000). Animal studies using the spontaneous diabetic rat model (the BB-DP rat) showed that prolonged exclusive breastfeeding decreased diabetes incidence by 40–50 % and associated with increased frequency of Treg cells and less pro-inflammatory cytokine secretion in the mesenteric lymph nodes (Brugman et al. 2009b). Furthermore, antibiotic treatment reduces the incidence in both the BB-DP rat and the NOD mouse model for spontaneous diabetes (Brugman et al. 2006; Schwartz et al. 2007). Interestingly, in the BB-DP rat, the composition of the microbiota before onset of disease differed between BB-DP rats that did and rats that did not develop diabetes, suggesting that microbial dysbiosis occurs prior to disease onset (Brugman et al. 2006). Likewise, several studies report an association between breast milk and protection against infection such as diarrhea, atopic diseases and asthma during childhood (Gdalevich et al. 2001a, b; Sachdev et al. 1991; van Odijk et al. 2003). Interestingly, a meta-analysis of 12 human studies showed that the protective effect in most studies correlated with the (high) concentrations of transforming growth factor (TGF)-β1 or TGF-β2 in the milk (Oddy and Rosales 2010). A recent meta-analysis of studies published between 1983 and 2012 on breastfeeding and asthma in children reported a strong protective association at ages 0–2 years between breastfeeding and asthma, which diminished over time (Dogaru et al. 2014a, b). The availability of nutrients, and especially of milk oligosaccharides, in the intestinal tract of newborns also has a profound influence on the microbial species that are able to survive there. Indeed, it has been shown that breastfeeding and bottle feeding result in different microbial colonization patterns, which results in different host immune responses (Schwartz et al. 2012).

To improve the composition of infant formulas for mothers that cannot provide breastfeeding to their child, investigators try to develop formulas that resemble the composition of human breast milk. Recent developments include the use of prebiotics to provide non-digestible oligosaccharides and probiotics. Like breast milk, bovine milk also contains several proteins that have an immunomodulatory function such as large quantities of immunoglobulins, lactoferrin, caseins and cytokines like TGF-β, but only very low levels of oligosaccharides (van Neerven et al. 2012). Many of these proteins are, surprisingly, active across the species barrier. The active form of bovine TGF-β2 (the predominant cytokine in milk) is even 100 % identical to human TGF-β2, and bovine IL-10 is fully comparable to human IL-10 in its anti-inflammatory effects of human monocytes and dendritic cells (Chatterton et al. 2013; den Hartog et al. 2011). Bovine IgG can bind to human Fc gamma receptors on monocytes and neutrophils (den Hartog et al. 2014; Kramski et al. 2012), and bovine IgG recognizes a wide range of pathogens that can also infect humans such as respiratory syncytial virus (den Hartog et al. 2014; Xu et al. 2006). Bovine colostrum, that is extremely rich in bovine IgG, has been shown to significantly reduce recurrent diarrhea in AIDS patients, showing that bovine IgG can have an anti-pathogenic effect in humans (Floren et al. 2006). Milk also contains anti-microbial proteins, most prominently lactoferrin, lactoperoxidase and lysozyme. Lactoferrin was shown to protect low birth weight infants against necrotizing enterocolitis (Manzoni et al. 2014). In line with this, it has already been known for a long time that growing up in a farm environment lowers the risk of developing allergies (von Mutius 2012). Next to exposure to farm animals, drinking farm milk has also been implicated as a factor that might reduce allergy risk (Loss et al. 2012; van Neerven et al. 2012; van Neerven 2014). A recent study showed that consumption of raw milk inversely associated with development of rhinitis, respiratory tract infections, otitis, and fever in infants (Loss et al. 2015). However, since bovine milk is heated and homogenized, a substantial proportion of these protective proteins will be denatured in milk products (van Neerven 2014). New insights into how dietary components influence host immunity, continuously promote the development of health-stimulating or disease-preventing (infant) nutrition.

### Fermentation Products: How Bacterial Products Influence Host Immunity

The microbes that are present in the intestinal tract of mammals are important for digestion of foods that would otherwise not be available to the host. The products of bacterial fermentation, such as butyrate, are readily taken up by colonocytes for energy, but also have important immunological effects. Most of the bacteria that reside in the mammalian gastrointestinal tract are saccharolytic, meaning that they mainly feed on carbohydrates (Cummings and Macfarlane 1991). Human milk oligosaccharides are complex glycan molecules that are present in very high concentrations in breast milk. Several studies have shown that milk oligosaccharides influence the composition of the intestinal microbiota (Bode 2009; Gauhe et al. 1954; LoCascio et al. 2007). Human milk oligosaccharides promote the growth of *Bifidobacteria* (Gauhe et al. 1954; LoCascio et al. 2007), and prevent pathogenic bacterial adherence to epithelial cells by acting as a soluble ligand for glycan receptors (Hong et al. 2009; Lomax and Calder 2009; Naarding et al. 2005; van Liempt et al. 2006). Next to effects on the microbiota milk oligosaccharides and non-digestible carbohydrates have also been show to directly influence host immunity and epithelial cell biology (reviewed in Vos et al. 2007).

Short chain fatty acids (SCFAs) are the end products generated by the colonic microbiota (Macfarlane and Macfarlane 2003). The type of SCFA formed is dependent on the substrate provided. Acetate and butyrate are mainly the result of starch fermentation, while acetate is the end product from the fermentation of pectin and xylan (Englyst et al. 1987). The succinate and acrylate pathways have been shown to lead to propionate production (Flint et al. 2012; Macy and Probst 1979; Seeliger et al. 2002; Watanabe et al. 2012), and some bacteria can produce propionate from deoxy sugars such as fucose and rhamnose or lactate (Saxena et al. 2010). SCFAs can interact with G protein coupled receptors (GPR43, GPR41 and GPR109a) (Brown et al. 2003). GPR43 is mainly located on neutrophils, and at lower levels on PBMCs and monocytes, while GPR41 is expressed on PBMCs but not on neutrophils, monocytes and dendritic cells. Both receptors have also been found on intestinal epithelial cells, and recently it has been shown that binding of SCFAs to these G protein coupled receptors can promote inflammatory responses in mice. Binding of SCFAs to GPR43 and GPR41 induced colon epithelial cell production of chemokines, recruited leukocytes and activated effector T cells (Kim et al. 2013). Niacin receptor GPR109A has recently been shown to also be a receptor for butyrate in the colon. Singh et al. (Singh et al. 2014) reported that Gpr109a signaling induced differentiation of Tregs and IL-10 producing T cells through effects on colonic macrophages and dendritic cells. Both propionate and acetate can reduce LPS-induced TNF-α release from human neutrophils (Tedelind et al. 2007), and butyrate seems to inhibit chemotactic effects on human monocytes (Meijer et al. 2010). Furthermore, SCFAs have been shown to reduce cell adhesion thereby preventing immune cell infiltration (Miller et al. 2005); (Zapolska-Downar and Naruszewicz 2009). Interestingly, butyrate can inhibit histone deacetylase (HDAC). HDACs prevent gene transcription by keeping the chromatin in a closed form, so transcription is prevented. Butyrate inhibits this effect leading to hyper-acetylation and open chromatin (Davie 2003). Butyrate has been reported to have anti-inflammatory effect through its HDAC activity on the NF-κB pathway, IL-5 expression and COX-2 expression (Han et al. 2007; Park et al. 2004; Quivy and Van Lint 2004). Another interesting example of the effect of butyrate on host immunity comes from the study by Atarashi et al. (2013). They isolated 17 strains *Clostridial* species that were able to enhance Treg frequency and induce ICOS upon inoculation into germ-free mice (Atarashi et al. 2013). In a follow-up study of the same research group, they showed that these *Clostridiales* (indirectly or directly) induced butyrate that subsequently induced functional colonic Treg cells, via epigenetic modification of the Foxp3 gene in T cells (Furusawa et al. 2013).

In conclusion, SCFAs are able to modify host immunity directly by binding to receptors on host cells or indirectly through epigenetic changes of host DNA. These modifications result in activation or repression of host immune genes and the outcome will depend on the type of SCFA and host (immune) cell type studied. Whether SCFAs can induce epigenetic changes in the host throughout life or whether a specific window (early in life) exists is currently unknown.

### Vitamin A and D

Vitamin D deficiency together with vitamin A deficiency are two of the most common food-related medical conditions worldwide. As vitamin A and D are conveyed to the newborn via breast milk, vitamin A and D status of the mother is very important for the developing child. Vitamin D deficiency leads to poor skeletal development and bone and joint deterioration, while vitamin A deficiency is one of the important causes of blindness in children (Khan et al. 2007; Wong et al. 2014). Appropriate vitamin D status has been reported to convey protection against several cancers, bacterial infections and autoimmune diseases such as rheumatoid arthritis and multiple sclerosis (Glade 2013). Also, low vitamin D levels during pregnancy associates with increased risk for type 1 diabetes in the offspring. However, too much vitamin D (especially D2) might lead to local tissue intoxication (reviewed in Glade 2013). In recent years, vitamin A and D have received a lot of attention from immunologists. Vitamin A can be converted into retinal and subsequently into retinoic acid by dendritic cells and epithelial cells. In an elegant paper, Coombes et al. (2007) showed that in mice, retinoic acid together with TGF-β are essential for promotion of Tregs by CD103^+^ DCs. Recently, it was also shown that retinoic acid can promote the development of human CD103^+^ dendritic cells from monocytes (den Hartog et al. 2013). The CD103^+^ intestinal DC subset can convert retinal into retinoic acid because it expresses the retinal dehydrogenase enzymes (RALDH1 and RALDH2) (Coombes et al. 2007). Retinoic acid has been shown to inhibit Th17 and the conversion of Tregs into T follicular helper cells, and induce intestinal mucosal homing molecules CCR9 and α_4_β_7_ (Benson et al. 2007; Iwata et al. 2004; Mora et al. 2003; Mucida et al. 2007; Sun et al. 2007; Takahashi et al. 2012). Also, retinoic acid is important for IgA-secreting cells, since mice deficient for vitamin A lack these cells in the small intestine (Mora et al. 2006). There have been reports that miR-10a, a microRNA induced by retinoic acid in Th17 cells can induce expression of T-bet (associated with Th1 cells) (Takahashi et al. 2012). This indicates that next to Tregs, retinoic acid might also induce Th1 cells. Indeed, in an inflammatory environment retinoic acid could induce Th1 immunity (DePaolo et al. 2011). Vitamin A uptake via the diet, does not only influence the immune system of the mother, but also influences the fetal immune system. As shown by van de Pavert et al. (2014), pups derived from mice fed vitamin A-deficient diets had markedly reduced lymph node size and decreased efficiency of immune responses.

Vitamin D has been reported to enhance regulatory T cell induction via binding of the VDR-RXR (vitamin D receptor-retinoic X receptor) binding to an enhancer in the Foxp3 gene (Kang et al. 2012). While vitamin D deficiency causes a reduction in CD8αα^+^ intraepithelial T cells (Bruce and Cantorna 2011). Recently, Spencer et al. (2014) showed that vitamin A deficiency leads to severely diminished type 3 innate lymphoid cells (ILC3s), which results in compromised immunity to acute bacterial infection. Additionally, vitamin A deprivation resulted in increased IL-13-producing ILC2s and resistance to nematode infection in mice (Spencer et al. 2014). Since vitamins A and D can have several direct and indirect effects on cells and signaling pathways, further research is necessary to understand their complete role in immune modulation. These findings, however, suggest that exposure to certain dietary factors (both in mother and child) can have profound influence on the development and effectiveness of the immune response. As with many multi-factorial diseases, the interplay between host, microbes and dietary exposure might be different in each individual patient, making it extremely difficult to find causal relations rather than incidental associations. This is very well illustrated by what is known for inflammatory bowel disease (IBD).

## When Homeostasis between Host and Microbes is Lost: the Case of IBD

In recent years, genome-wide association studies have revealed many single nucleotide polymorphisms (SNPs) in host genes that are associated with multi-factorial diseases. For example, in IBD >160 genes are found to be associated with either ulcerative colitis and Crohn’s disease or both (Ventham et al. 2013). Each and every patient, therefore, can have a unique combinations of these SNPs. Interestingly, several of these associated genes has a role in bacterial–host interaction. Studies performed using experimental animals showed that knock-outs of these genes (such as Nod2 or enteric defensins) can change the intestinal microbial community (Salzman et al. 2010; Secher et al. 2013). Subsequently, changes in microbial community can influence disease susceptibility. The IL-10 knock-out mice, for example, does not develop colitis under germ-free conditions. Interestingly, narrow and broad spectrum antibiotics can prevent disease in IL-10^−/−^ mice under specific pathogen-free conditions (Hoentjen et al. 2003). Furthermore, we have shown that that the composition of zebrafish intestinal microbiota can determine recruitment of different immune cells, enterocolitis susceptibility and severity (Brugman et al. 2009a).

An illustration of influence of gene alterations on microbial dysbiosis and disease susceptibility comes from the studies performed by Garrett et al. (2007). Mice deficient for transcription factor T-bet and Rag2 (TRUC mice) showed increased TNF-α production by colonic dendritic cells leading to increased apoptosis of colonic epithelial cells and spontaneous colitis. This colitis was dependent on the intestinal microbiota since treatment of TRUC mice with a combination of antibiotics cured the mice from colitis. Later studies confirmed that TRUC mice have an altered microbiota (presence of *Klebsiella pneumoniae* and *Proteus mirabilis*) (Garrett et al. 2010). This colitis was also transmissible via the microbiota, since co-housing adult TRUC mice and wild-type (WT) mice (3:1) rendered WT mice more susceptible to develop colitis. Likewise, when a TRUC mother fostered pups of Rag2^−/−^ or WT mice, these mice pups were also more susceptible and developed colitis that was histologically similar to colitis in TRUC mice (Garrett et al. 2010).

Another study that illustrates the importance of a functioning adaptive immune system was performed using zebrafish. In zebrafish, lymphocytes deficiency leads to failure to suppress bacteria of the order *Vibrionales* (that contains known fish pathogens) (our own unpublished observations). Adoptive transfer of T lymphocytes could actively suppress outgrowth of these *Vibrionales*. Additionally, zebrafish T lymphocytes are able to induce epithelial *Cxcl8*-*l1* expression, thereby augmenting mucosal immune responses (Brugman et al. 2014). In summary, these studies emphasize that genetic deficiencies (of genes involved in mucosal immunity) can modify the mucosal environment and allow for modulation of the microbiota which in turn can alter susceptibility towards disease. This clearly illustrates that modulation of the gut microbiota might be beneficial for IBD patients. Indeed, Sokol et al. (2008, 2009) identified *Faecalibacterium prausnitzii* as an anti-inflammatory commensal bacterium, which was severely reduced in Crohn disease patients. These studies have encouraged fecal transplantation as a therapy for IBD patients, which results in remission in some but not all patients (Angelberger et al. 2013; Kao et al. 2014; Rubin 2013). Clearly, future research to elucidate the complex interaction between host, diet and microbes in the context of chronic intestinal inflammation and during health is dearly needed.

## Timing of Exposure, Does a Window of Opportunity Exist?

Next to investigating the different pathways by which food and microbes alter host immunity, investigation on the concept of timing will be crucial. It has been suggested, that a window exists early in life when microbes alter host immunity, after which a set point is reached and homeostasis is established. There is indication that some processes might indeed take place in a specific time window, where after they cannot be changed again. For example, invariant natural killer T cells (iNKT) cells, a subset of invariant T cells that recognize glycolipids in the context of MHC-like molecule CD1d, were found to be more abundant in the colon (and lungs) of germ-free mice (Olszak et al. 2012). These germ-free mice displayed increased morbidity in models of IBD and allergic asthma. The increased number of iNKT cells in the colon (and lungs) of germ-free mice was shown to be the result of high expression of the chemokine CXCL16. Colonization of neonatal—but not adult—germ-free mice protected the animals from this mucosal iNKT accumulation and related pathology (Olszak et al. 2012). This difference in iNKT accumulation associated with epigenetic modifications that enabled modification of CXCL16 expression early in life, but not at adult age. This suggests that a host developmental (epigenetic) program exists that allows for environmental agents to shape immune responses only at certain time points of life. However, other studies suggest microbial and dietary modulation can also affect host immunity in later life. The success of fecal transplants in obese people and inflammatory bowel disease patients suggests that lifelong modification of diet and microbes might be beneficial (Smits et al. 2013). Likewise, it has been shown that glucosinolates derived from vegetables in the diet, such as cabbage and broccoli, can activate the aryl hydrocarbon receptor (AhR) and modulate immune responses (Bjeldanes et al. 1991). AhR ligand TCDD can induce differentiation of Tregs while inhibiting development of Th17 cells, which correlates with increased methylation and demethylation of the respective promoters for Foxp3 and IL-17, indicating that epigenetic modification can occur upon AhR activation (Singh et al. 2011). Thus, whereas host epigenetic changes might be induced by bacteria or nutrients, it is not clear whether a specific window (early) in life exists or whether it can take place throughout life.

### Future Perspectives

**Fig. 2:**
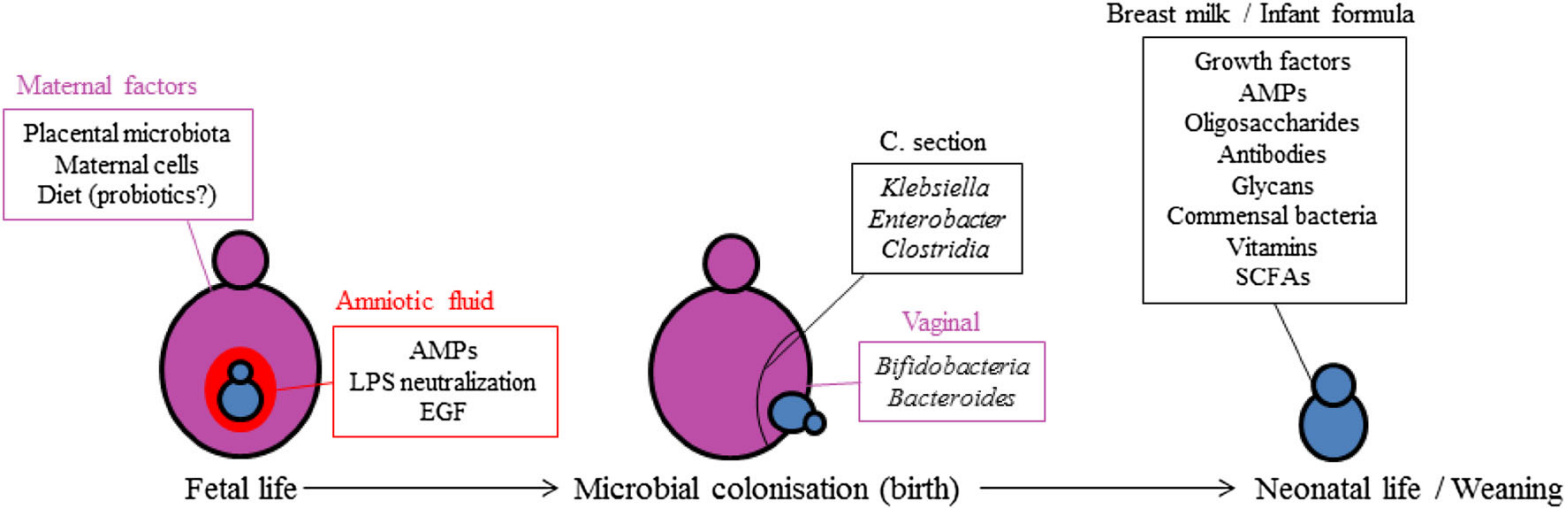
Important factors in early life affecting mucosal immune development. During the fetal life stage, there is a direct interaction between maternally derived environmental factors (e.g., diet and microbes) and the fetus. Additionally, the amniotic fluid contains anti-microbial peptides (AMPs) and epidermal growth factors (EGF) and endotoxin-neutralizing proteins that protect against pathogenic bacteria and possible fatal immune responses, respectively. Birth, and the way of delivery, is a critical point in immune development that determines which types of microbes will colonize the GI-tract. In the neonatal life stage, breast milk (or alternatively infant formula) provides the infant with proteins, short chain fatty acids (SCFAs) and vitamins that are critical for immune cell differentiation and development. Environmental factors such as diet and microbes early in life set a immunological stage that impacts the hosts susceptibility towards disease

In the last decade, through the development of large-scale metagenomic technologies, we have gained access to enormous datasets containing information on microbial and host genes in health and disease. The future challenge will be to make sense of these large datasets and to stratify patient groups according to their genomic or metabolomic profiles. In addition, modification of the mucosal immune system through dietary interventions (in both mothers and infants) requires more in depth knowledge on how dietary nutrients or microbial patterns can alter host immunity (Fig. 2). The fact that fetal life might not be as devoid of environmental stimulation as previously thought suggests that modification of the environment during pregnancy and early life might be able to (beneficially) alter immunity. Furthermore, epigenetic modification of the host by bacteria or dietary components might be time dependent. Future research should focus on the question whether host epigenetic modification can only be achieved in a specific window (early) in life or whether changes can be induced lifelong. Rapid technological advances in this field as evidenced by large metagenomic screens and epigenetic sequencing platforms will soon provide more answers on these questions.

In conclusion, environmental factors, such as dietary components and microbes can shape the mucosal immune system by influencing differentiation and development of immune cells and tissues. This in turn influences host susceptibility towards disease. By using model systems that can be easily manipulated both genetically and environmentally (i.e., zebrafish and mice) novel pathways can be discovered that control host responses towards environmental antigens. Elucidation of these conserved pathways will yield novel targets for nutritional interventions that will benefit human health.

## References

[CR1] Aagaard K, Ma J, Antony KM (2014). The placenta harbors a unique microbiome. Sci Transl Med.

[CR2] Adlerberth I, Carlsson B, de Man P (1991). Intestinal colonization with Enterobacteriaceae in Pakistani and Swedish hospital-delivered infants. Acta Paediatr Scand.

[CR3] Adlerberth I, Lindberg E, Aberg N (2006). Reduced enterobacterial and increased staphylococcal colonization of the infantile bowel: an effect of hygienic lifestyle?. Pediatr Res.

[CR4] Adlerberth I, Strachan DP, Matricardi PM (2007). Gut microbiota and development of atopic eczema in 3 European birth cohorts. J Allergy Clin Immunol.

[CR5] Angelberger S, Reinisch W, Makristathis A (2013). Temporal bacterial community dynamics vary among ulcerative colitis patients after fecal microbiota transplantation. Am J Gastroenterol.

[CR6] Atarashi K, Tanoue T, Oshima K (2013). Treg induction by a rationally selected mixture of Clostridia strains from the human microbiota. Nature.

[CR7] Bager P, Wohlfahrt J, Westergaard T (2008). Caesarean delivery and risk of atopy and allergic disease: meta-analyses. Clin Exp Allergy.

[CR8] Benson MJ, Pino-Lagos K, Rosemblatt M (2007). All-trans retinoic acid mediates enhanced T reg cell growth, differentiation, and gut homing in the face of high levels of co-stimulation. J Exp Med.

[CR9] Bettelli E, Oukka M, Kuchroo VK (2007). T(H)-17 cells in the circle of immunity and autoimmunity. Nat Immunol.

[CR10] Bevins CL, Salzman NH (2011). Paneth cells, antimicrobial peptides and maintenance of intestinal homeostasis. Nat Rev Microbiol.

[CR11] Biasucci G, Rubini M, Riboni S (2010). Mode of delivery affects the bacterial community in the newborn gut. Early Hum Dev.

[CR12] Bjeldanes LF, Kim JY, Grose KR (1991). Aromatic hydrocarbon responsiveness-receptor agonists generated from indole-3-carbinol in vitro and in vivo: comparisons with 2,3,7,8-tetrachlorodibenzo-p-dioxin. Proc Natl Acad Sci USA.

[CR13] Bode L (2009). Human milk oligosaccharides: prebiotics and beyond. Nutr Rev.

[CR14] Borch-Johnsen K, Joner G, Mandrup-Poulsen T (1984). Relation between breast-feeding and incidence rates of insulin-dependent diabetes mellitus. A hypothesis. Lancet.

[CR15] Boyle RJ, Mah LJ, Chen A (2008). Effects of Lactobacillus GG treatment during pregnancy on the development of fetal antigen-specific immune responses. Clin Exp Allergy.

[CR16] Breel M, Van der Ende M, Sminia T (1988). Subpopulations of lymphoid and non-lymphoid cells in bronchus-associated lymphoid tissue (BALT) of the mouse. Immunology.

[CR17] Breel M, van der Ende MB, Sminia T (1988). Subpopulations of non-lymphoid cells in bronchus associated lymphoid tissue and lung of the mouse. Adv Exp Med Biol.

[CR18] Brown AJ, Goldsworthy SM, Barnes AA (2003). The Orphan G protein-coupled receptors GPR41 and GPR43 are activated by propionate and other short chain carboxylic acids. J Biol Chem.

[CR19] Bruce D, Cantorna MT (2011). Intrinsic requirement for the vitamin D receptor in the development of CD8alphaalpha-expressing T cells. J Immunol.

[CR20] Brugman S, Klatter FA, Visser JT (2006). Antibiotic treatment partially protects against type 1 diabetes in the Bio-Breeding diabetes-prone rat. Is the gut flora involved in the development of type 1 diabetes?. Diabetologia.

[CR21] Brugman S, Liu KY, Lindenbergh-Kortleve D (2009). Oxazolone-induced enterocolitis in zebrafish depends on the composition of the intestinal microbiota. Gastroenterology.

[CR22] Brugman S, Visser JT, Hillebrands JL (2009). Prolonged exclusive breastfeeding reduces autoimmune diabetes incidence and increases regulatory T-cell frequency in bio-breeding diabetes-prone rats. Diabetes Metab Res Rev.

[CR23] Brugman S, Witte M, Scholman RC (2014). T lymphocyte-dependent and -independent regulation of Cxcl8 expression in zebrafish intestines. J Immunol.

[CR24] Byrne JA, Stankovic AK, Cooper MD (1994). A novel subpopulation of primed T cells in the human fetus. J Immunol.

[CR25] Cebra JJ (1999). Influences of microbiota on intestinal immune system development. Am J Clin Nutr.

[CR26] Chatterton DE, Nguyen DN, Bering SB (2013). Anti-inflammatory mechanisms of bioactive milk proteins in the intestine of newborns. Int J Biochem Cell Biol.

[CR27] Cherry SH, Filler M, Harvey H (1973). Lysozyme content of amniotic fluid. Am J Obstet Gynecol.

[CR28] Clark JA, Doelle SM, Halpern MD (2006). Intestinal barrier failure during experimental necrotizing enterocolitis: protective effect of EGF treatment. Am J Physiol Gastrointest Liver Physiol.

[CR29] Coombes JL, Siddiqui KR, Arancibia-Carcamo CV (2007). A functionally specialized population of mucosal CD103 + DCs induces Foxp3 + regulatory T cells via a TGF-beta and retinoic acid-dependent mechanism. J Exp Med.

[CR30] Coppa GV, Bruni S, Morelli L (2004). The first prebiotics in humans: human milk oligosaccharides. J Clin Gastroenterol.

[CR31] Corbett AJ, Eckle SB, Birkinshaw RW (2014). T-cell activation by transitory neo-antigens derived from distinct microbial pathways. Nature.

[CR32] Couper JJ, Steele C, Beresford S (1999). Lack of association between duration of breast-feeding or introduction of cow’s milk and development of islet autoimmunity. Diabetes.

[CR33] Crabbe PA, Bazin H, Eyssen H (1968). The normal microbial flora as a major stimulus for proliferation of plasma cells synthesizing IgA in the gut. The germ-free intestinal tract. Int Arch Allergy Appl Immunol.

[CR34] Crellin NK, Trifari S, Kaplan CD (2010). Human NKp44+IL-22+cells and LTi-like cells constitute a stable RORC+ lineage distinct from conventional natural killer cells. J Exp Med.

[CR35] Cummings JH, Macfarlane GT (1991). The control and consequences of bacterial fermentation in the human colon. J Appl Bacteriol.

[CR36] Cupedo T, Nagasawa M, Weijer K (2005). Development and activation of regulatory T cells in the human fetus. Eur J Immunol.

[CR37] Darrasse-Jeze G, Marodon G, Salomon BL (2005). Ontogeny of CD4+CD25+ regulatory/suppressor T cells in human fetuses. Blood.

[CR38] Davie JR (2003). Inhibition of histone deacetylase activity by butyrate. J Nutr.

[CR39] de Oliveira IR, de Araujo AN, Bao SN (2001). Binding of lactoferrin and free secretory component to enterotoxigenic *Escherichia coli*. FEMS Microbiol Lett.

[CR40] de Roock S, Stoppelenburg AJ, Scholman R (2013). Defective TH17 development in human neonatal T cells involves reduced RORC2 mRNA content. J Allergy Clin Immunol.

[CR41] den Hartog G, Savelkoul HF, Schoemaker R (2011). Modulation of human immune responses by bovine interleukin-10. PLoS One.

[CR42] den Hartog G, van Altena C, Savelkoul HF (2013). The mucosal factors retinoic acid and TGF-beta1 induce phenotypically and functionally distinct dendritic cell types. Int Arch Allergy Immunol.

[CR43] den Hartog G, Jacobino S, Bont L (2014). Specificity and effector functions of human RSV-specific IgG from bovine milk. PLoS One.

[CR44] DePaolo RW, Abadie V, Tang F (2011). Co-adjuvant effects of retinoic acid and IL-15 induce inflammatory immunity to dietary antigens. Nature.

[CR45] Dogaru CM, Nyffenegger D, Pescatore AM (2014). Breastfeeding and childhood asthma: systematic review and meta-analysis. Am J Epidemiol.

[CR46] Dogaru CM, Nyffenegger D, Pescatore AM (2014). Dogaru et al. respond to “Does breastfeeding protect against ‘asthma’?”. Am J Epidemiol.

[CR47] Dominguez-Bello MG, Costello EK, Contreras M (2010). Delivery mode shapes the acquisition and structure of the initial microbiota across multiple body habitats in newborns. Proc Natl Acad Sci USA.

[CR48] Duchmann R, Kaiser I, Hermann E (1995). Tolerance exists towards resident intestinal flora but is broken in active inflammatory bowel disease (IBD). Clin Exp Immunol.

[CR49] Eggesbo M, Botten G, Stigum H (2003). Is delivery by cesarean section a risk factor for food allergy?. J Allergy Clin Immunol.

[CR50] Englyst HN, Trowell H, Southgate DA (1987). Dietary fiber and resistant starch. Am J Clin Nutr.

[CR51] Espinoza J, Romero R, Chaiworapongsa T (2002). Lipopolysaccharide-binding protein in microbial invasion of the amniotic cavity and human parturition. J Matern Fetal Neonatal Med.

[CR52] Fallani M, Young D, Scott J (2010). Intestinal microbiota of 6-week-old infants across Europe: geographic influence beyond delivery mode, breast-feeding, and antibiotics. J Pediatr Ggastroenterol Nutr.

[CR53] Fanaro S, Chierici R, Guerrini P (2003). Intestinal microflora in early infancy: composition and development. Acta Paediatr Suppl.

[CR54] Flint HJ, Scott KP, Duncan SH (2012). Microbial degradation of complex carbohydrates in the gut. Gut Microbes.

[CR55] Floren CH, Chinenye S, Elfstrand L (2006). ColoPlus, a new product based on bovine colostrum, alleviates HIV-associated diarrhoea. Scand J Gastroenterol.

[CR56] Furusawa Y, Obata Y, Fukuda S (2013). Commensal microbe-derived butyrate induces the differentiation of colonic regulatory T cells. Nature.

[CR57] Gaboriau-Routhiau V, Rakotobe S, Lecuyer E (2009). The key role of segmented filamentous bacteria in the coordinated maturation of gut helper T cell responses. Immunity.

[CR58] Garrett WS, Lord GM, Punit S (2007). Communicable ulcerative colitis induced by T-bet deficiency in the innate immune system. Cell.

[CR59] Garrett WS, Gallini CA, Yatsunenko T (2010). Enterobacteriaceae act in concert with the gut microbiota to induce spontaneous and maternally transmitted colitis. Cell Host Microbe.

[CR60] Gauhe A, Gyorgy P, Hoover JR (1954). Bifidus factor. IV. Preparations obtained from human milk. Arch Biochem Biophys.

[CR61] Gdalevich M, Mimouni D, David M (2001). Breast-feeding and the onset of atopic dermatitis in childhood: a systematic review and meta-analysis of prospective studies. J Am Acad Dermatol.

[CR62] Gdalevich M, Mimouni D, Mimouni M (2001). Breast-feeding and the risk of bronchial asthma in childhood: a systematic review with meta-analysis of prospective studies. J Pediatr.

[CR63] Giugliano LG, Ribeiro ST, Vainstein MH (1995). Free secretory component and lactoferrin of human milk inhibit the adhesion of enterotoxigenic *Escherichia coli*. J Med Microbiol.

[CR64] Glade MJ (2013). Vitamin D: health panacea or false prophet?. Nutrition.

[CR65] Good M, Siggers RH, Sodhi CP (2012). Amniotic fluid inhibits Toll-like receptor 4 signaling in the fetal and neonatal intestinal epithelium. Proc Natl Acad Sci USA.

[CR66] Hall MA, Cole CB, Smith SL (1990). Factors influencing the presence of faecal lactobacilli in early infancy. Arch Dis Child.

[CR67] Halpern MD, Dominguez JA, Dvorakova K (2003). Ileal cytokine dysregulation in experimental necrotizing enterocolitis is reduced by epidermal growth factor. J Pediatr Gastroenterol Nutr.

[CR68] Hameleers DM, Stoop AE, van der Ven I (1989). Intra-epithelial lymphocytes and non-lymphoid cells in the human nasal mucosa. Int Arch Allergy Appl Immunol.

[CR69] Hammerschmidt S, Bethe G, Remane PH (1999). Identification of pneumococcal surface protein A as a lactoferrin-binding protein of *Streptococcus pneumoniae*. Infect Immun.

[CR70] Han S, Lu J, Zhang Y (2007). HDAC inhibitors TSA and sodium butyrate enhanced the human IL-5 expression by altering histone acetylation status at its promoter region. Immunol Lett.

[CR71] Hapfelmeier S, Lawson MA, Slack E (2010). Reversible microbial colonization of germ-free mice reveals the dynamics of IgA immune responses. Science.

[CR72] Hasnain SZ, Gallagher AL, Grencis RK (2013). A new role for mucins in immunity: insights from gastrointestinal nematode infection. Int J Biochem Cell Biol.

[CR73] Haynes BF, Martin ME, Kay HH (1988). Early events in human T cell ontogeny. Phenotypic characterization and immunohistologic localization of T cell precursors in early human fetal tissues. J Exp Med.

[CR74] Hayward AR, Ezer G (1974). Development of lymphocyte populations in the human foetal thymus and spleen. Clin Exp Immunol.

[CR75] Heikkila MP, Saris PE (2003). Inhibition of *Staphylococcus aureus* by the commensal bacteria of human milk. J Appl Microbiol.

[CR76] Hoentjen F, Harmsen HJ, Braat H (2003). Antibiotics with a selective aerobic or anaerobic spectrum have different therapeutic activities in various regions of the colon in interleukin 10 gene deficient mice. Gut.

[CR77] Holgerson PL, Vestman NR, Claesson R (2013). Oral microbial profile discriminates breast-fed from formula-fed infants. J Pediatr Gastroenterol Nutr.

[CR78] Hong P, Ninonuevo MR, Lee B (2009). Human milk oligosaccharides reduce HIV-1-gp120 binding to dendritic cell-specific ICAM3-grabbing non-integrin (DC-SIGN). Br J Nutr.

[CR79] Hummel M, Fuchtenbusch M, Schenker M (2000). No major association of breast-feeding, vaccinations, and childhood viral diseases with early islet autoimmunity in the German BABYDIAB Study. Diabetes Care.

[CR80] Hunt KM, Foster JA, Forney LJ (2011). Characterization of the diversity and temporal stability of bacterial communities in human milk. PLoS One.

[CR81] Husband AJ, Gleeson M (1996). Ontogeny of mucosal immunity–environmental and behavioral influences. Brain Behav Immun.

[CR82] Huurre A, Kalliomaki M, Rautava S (2008). Mode of delivery—effects on gut microbiota and humoral immunity. Neonatology.

[CR83] Ivanov II, Atarashi K, Manel N (2009). Induction of intestinal Th17 cells by segmented filamentous bacteria. Cell.

[CR84] Iwata M, Hirakiyama A, Eshima Y (2004). Retinoic acid imprints gut-homing specificity on T cells. Immunity.

[CR85] Jimenez E, Fernandez L, Marin ML (2005). Isolation of commensal bacteria from umbilical cord blood of healthy neonates born by cesarean section. Curr Microbiol.

[CR86] Jimenez E, Marin ML, Martin R (2008). Is meconium from healthy newborns actually sterile?. Res Microbiol.

[CR87] Kang SW, Kim SH, Lee N (2012). 1,25-Dihyroxyvitamin D3 promotes FOXP3 expression via binding to vitamin D response elements in its conserved noncoding sequence region. J Immunol.

[CR88] Kao D, Hotte N, Gillevet P (2014). Fecal microbiota transplantation inducing remission in Crohn’s colitis and the associated changes in fecal microbial profile. J Clin Gastroenterol.

[CR89] Khan NC, West CE, de Pee S (2007). The contribution of plant foods to the vitamin A supply of lactating women in Vietnam: a randomized controlled trial. Am J Clin Nutr.

[CR90] Kim YS, Ho SB (2010). Intestinal goblet cells and mucins in health and disease: recent insights and progress. Curr Gastroenterol Rep.

[CR91] Kim HS, Cho JH, Park HW (2002). Endotoxin-neutralizing antimicrobial proteins of the human placenta. J Immunol.

[CR92] Kim MH, Kang SG, Park JH (2013). Short-chain fatty acids activate GPR41 and GPR43 on intestinal epithelial cells to promote inflammatory responses in mice. Gastroenterology.

[CR93] Kiss EA, Vonarbourg C, Kopfmann S (2011). Natural aryl hydrocarbon receptor ligands control organogenesis of intestinal lymphoid follicles. Science.

[CR94] Kjer-Nielsen L, Patel O, Corbett AJ (2012). MR1 presents microbial vitamin B metabolites to MAIT cells. Nature.

[CR95] Kosaka N, Izumi H, Sekine K (2010). microRNA as a new immune-regulatory agent in breast milk. Silence.

[CR96] Kramski M, Center RJ, Wheatley AK (2012). Hyperimmune bovine colostrum as a low-cost, large-scale source of antibodies with broad neutralizing activity for HIV-1 envelope with potential use in microbicides. Antimicro Agents Chemother.

[CR97] Laubereau B, Filipiak-Pittroff B, von Berg A (2004). Caesarean section and gastrointestinal symptoms, atopic dermatitis, and sensitisation during the first year of life. Arch Dis Child.

[CR98] Le Bourhis L, Martin E, Peguillet I (2010). Antimicrobial activity of mucosal-associated invariant T cells. Nat Immunol.

[CR99] LeBouder E, Rey-Nores JE, Rushmere NK (2003). Soluble forms of Toll-like receptor (TLR)2 capable of modulating TLR2 signaling are present in human plasma and breast milk. J Immunol.

[CR100] Lee JS, Cella M, Colonna M (2012). AHR and the transcriptional regulation of type-17/22 ILC. Front Immunol.

[CR101] Leeansyah E, Loh L, Nixon DF (2014). Acquisition of innate-like microbial reactivity in mucosal tissues during human fetal MAIT-cell development. Nat Commun.

[CR102] Li Y, Innocentin S, Withers DR (2011). Exogenous stimuli maintain intraepithelial lymphocytes via aryl hydrocarbon receptor activation. Cell.

[CR103] Ling JM, Schryvers AB (2006). Perspectives on interactions between lactoferrin and bacteria. Biochem Cell Biol.

[CR104] LoCascio RG, Ninonuevo MR, Freeman SL (2007). Glycoprofiling of bifidobacterial consumption of human milk oligosaccharides demonstrates strain specific, preferential consumption of small chain glycans secreted in early human lactation. J Agric Food Chem.

[CR105] Lomax AR, Calder PC (2009). Prebiotics, immune function, infection and inflammation: a review of the evidence. Br J Nutr.

[CR106] Loss G, Bitter S, Wohlgensinger J (2012). Prenatal and early-life exposures alter expression of innate immunity genes: the PASTURE cohort study. J Allergy Clin Immunol.

[CR107] Loss G, Depner M, Ulfman LH (2015). Consumption of unprocessed cow’s milk protects infants from common respiratory infections. J Allergy Clin Immunol.

[CR108] Macchiaverni P, Rekima A, Turfkruyer M (2014). Respiratory allergen from house dust mite is present in human milk and primes for allergic sensitization in a mouse model of asthma. Allergy.

[CR109] Macfarlane S, Macfarlane GT (2003). Regulation of short-chain fatty acid production. Proc Nutr Soc.

[CR110] Macpherson AJ, Uhr T (2004). Induction of protective IgA by intestinal dendritic cells carrying commensal bacteria. Science.

[CR111] Macpherson AJ, Geuking MB, McCoy KD (2005). Immune responses that adapt the intestinal mucosa to commensal intestinal bacteria. Immunology.

[CR112] Macy JM, Probst I (1979). The biology of gastrointestinal bacteroides. Annu Rev Microbiol.

[CR113] Malamitsi-Puchner A, Protonotariou E, Boutsikou T (2005). The influence of the mode of delivery on circulating cytokine concentrations in the perinatal period. Early Hum Dev.

[CR114] Maloy KJ, Powrie F (2011). Intestinal homeostasis and its breakdown in inflammatory bowel disease. Nature.

[CR115] Manzoni P, Meyer M, Stolfi I (2014). Bovine lactoferrin supplementation for prevention of necrotizing enterocolitis in very-low-birth-weight neonates: a randomized clinical trial. Early Hum Dev.

[CR116] Martin R, Jimenez E, Heilig H (2009). Isolation of bifidobacteria from breast milk and assessment of the bifidobacterial population by PCR-denaturing gradient gel electrophoresis and quantitative real-time PCR. Appl Environ Microbiol.

[CR117] Mayer EJ, Hamman RF, Gay EC (1988). Reduced risk of IDDM among breast-fed children. The Colorado IDDM Registry. Diabetes.

[CR118] Maynard CL, Elson CO, Hatton RD (2012). Reciprocal interactions of the intestinal microbiota and immune system. Nature.

[CR119] McKenzie BS, Kastelein RA, Cua DJ (2006). Understanding the IL-23-IL-17 immune pathway. Trends Immunol.

[CR120] Meijer K, de Vos P, Priebe MG (2010). Butyrate and other short-chain fatty acids as modulators of immunity: what relevance for health?. Curr Opin Clin Nutr Metab Care.

[CR121] Michaelsson J, Mold JE, McCune JM (2006). Regulation of T cell responses in the developing human fetus. J Immunol.

[CR122] Miller SJ, Zaloga GP, Hoggatt AM (2005). Short-chain fatty acids modulate gene expression for vascular endothelial cell adhesion molecules. Nutrition.

[CR123] Mold JE, Michaelsson J, Burt TD (2008). Maternal alloantigens promote the development of tolerogenic fetal regulatory T cells in utero. Science.

[CR124] Mora JR, Bono MR, Manjunath N (2003). Selective imprinting of gut-homing T cells by Peyer’s patch dendritic cells. Nature.

[CR125] Mora JR, Iwata M, Eksteen B (2006). Generation of gut-homing IgA-secreting B cells by intestinal dendritic cells. Science.

[CR126] Mucida D, Park Y, Kim G (2007). Reciprocal TH17 and regulatory T cell differentiation mediated by retinoic acid. Science.

[CR127] Naarding MA, Ludwig IS, Groot F (2005). Lewis X component in human milk binds DC-SIGN and inhibits HIV-1 transfer to CD4+T lymphocytes. J Clin Invest.

[CR128] Newburg DS (2005). Innate immunity and human milk. J Nutr.

[CR129] Newburg DS (2012). Prevention of rotavirus-induced diarrhea. J Pediatr Gastroenterol Nutr.

[CR130] Nieuwenhuis EE, Matsumoto T, Lindenbergh D (2009). Cd1d-dependent regulation of bacterial colonization in the intestine of mice. J Clin Invest.

[CR131] Oddy WH, Rosales F (2010). A systematic review of the importance of milk TGF-beta on immunological outcomes in the infant and young child. Pediatr Allergy Immunol.

[CR132] Olszak T, An D, Zeissig S (2012). Microbial exposure during early life has persistent effects on natural killer T cell function. Science.

[CR133] Oozeer R, van Limpt K, Ludwig T (2013). Intestinal microbiology in early life: specific prebiotics can have similar functionalities as human-milk oligosaccharides. Am J Clin Nutr.

[CR134] Ouyang W, Kolls JK, Zheng Y (2008). The biological functions of T helper 17 cell effector cytokines in inflammation. Immunity.

[CR135] Pabst R, Gehrke I (1990). Is the bronchus-associated lymphoid tissue (BALT) an integral structure of the lung in normal mammals, including humans?. Am J Respir Cell Mol Biol.

[CR136] Pabst R, Russell MW, Brandtzaeg P (2008). Tissue distribution of lymphocytes and plasma cells and the role of the gut. Trends Immunol.

[CR137] Park GY, Joo M, Pedchenko T (2004). Regulation of macrophage cyclooxygenase-2 gene expression by modifications of histone H3. American journal of physiology. Lung Cell Mol Physiol.

[CR138] Passali D (1992). Hypertrophy of adenoids and tubal functionality. Adv Otorhinolaryngol.

[CR139] Penders J, Thijs C, Vink C (2006). Factors influencing the composition of the intestinal microbiota in early infancy. Pediatrics.

[CR140] Penders J, Thijs C, van den Brandt PA (2007). Gut microbiota composition and development of atopic manifestations in infancy: the KOALA Birth Cohort Study. Gut.

[CR141] Penders J, Gerhold K, Stobberingh EE (2013). Establishment of the intestinal microbiota and its role for atopic dermatitis in early childhood. J Allergy Clin Immunol.

[CR142] Perry M, Whyte A (1998). Immunology of the tonsils. Immunol Today.

[CR143] Phalipon A, Cardona A, Kraehenbuhl JP (2002). Secretory component: a new role in secretory IgA-mediated immune exclusion in vivo. Immunity.

[CR144] Poulsen SS, Kryger-Baggesen N, Nexo E (1996). Immunohistochemical localization of epidermal growth factor in the second-trimester human fetus. Histochem Cell Biol.

[CR145] Qiu X, Zhang M, Yang X (2013). *Faecalibacterium prausnitzii* upregulates regulatory T cells and anti-inflammatory cytokines in treating TNBS-induced colitis. J Crohns Colitis.

[CR146] Quivy V, Van Lint C (2004). Regulation at multiple levels of NF-kappaB-mediated transactivation by protein acetylation. Biochem Pharmacol.

[CR147] Rautava S, Collado MC, Salminen S (2012). Probiotics modulate host-microbe interaction in the placenta and fetal gut: a randomized, double-blind, placebo-controlled trial. Neonatology.

[CR148] Rawls JF, Samuel BS, Gordon JI (2004). Gnotobiotic zebrafish reveal evolutionarily conserved responses to the gut microbiota. Proc Natl Acad Sci USA.

[CR149] Reynders A, Yessaad N, Vu Manh TP (2011). Identity, regulation and in vivo function of gut NKp46+RORgammat+ and NKp46+RORgammat- lymphoid cells. EMBO J.

[CR150] Rogier EW, Frantz AL, Bruno ME (2014). Secretory antibodies in breast milk promote long-term intestinal homeostasis by regulating the gut microbiota and host gene expression. Proc Natl Acad Sci USA.

[CR151] Rogosch T, Kerzel S, Hoss K (2012). IgA response in preterm neonates shows little evidence of antigen-driven selection. J Immunol.

[CR152] Rosenbauer J, Herzig P, Giani G (2008). Early infant feeding and risk of type 1 diabetes mellitus-a nationwide population-based case-control study in pre-school children. Diabetes Metab Res Rev.

[CR153] Round JL, Mazmanian SK (2010). Inducible Foxp3+ regulatory T-cell development by a commensal bacterium of the intestinal microbiota. Proc Natl Acad Sci USA.

[CR154] Rubin DT (2013). Curbing our enthusiasm for fecal transplantation in ulcerative colitis. Am J Gastroenterol.

[CR155] Sachdev HP, Kumar S, Singh KK (1991). Does breastfeeding influence mortality in children hospitalized with diarrhoea?. J Trop Pediatr.

[CR156] Salzman NH, Hung K, Haribhai D (2010). Enteric defensins are essential regulators of intestinal microbial ecology. Nat Immunol.

[CR157] Sanos SL, Bui VL, Mortha A (2009). RORgammat and commensal microflora are required for the differentiation of mucosal interleukin 22-producing NKp46+ cells. Nat Immunol.

[CR158] Satoh-Takayama N, Lesjean-Pottier S, Sawa S (2011). Lymphotoxin-beta receptor-independent development of intestinal IL-22-producing NKp46+ innate lymphoid cells. Eur J Immunol.

[CR159] Satokari R, Gronroos T, Laitinen K (2009). Bifidobacterium and Lactobacillus DNA in the human placenta. Lett Appl Microbiol.

[CR160] Saxena RK, Anand P, Saran S (2010). Microbial production and applications of 1,2-propanediol. Indian J Microbiol.

[CR161] Scholz-Ahrens KE, Schrezenmeir J (2007). Inulin and oligofructose and mineral metabolism: the evidence from animal trials. J Nutr.

[CR162] Scholz-Ahrens KE, Ade P, Marten B (2007). Prebiotics, probiotics, and synbiotics affect mineral absorption, bone mineral content, and bone structure. J Nutr.

[CR163] Schwartz RF, Neu J, Schatz D (2007). Comment on: Brugman S et al. (2006) Antibiotic treatment partially protects against type 1 diabetes in the Bio-Breeding diabetes-prone rat. Is the gut flora involved in the development of type 1 diabetes?. Diabetologia.

[CR164] Schwartz S, Friedberg I, Ivanov IV (2012). A metagenomic study of diet-dependent interaction between gut microbiota and host in infants reveals differences in immune response. Genome Biol.

[CR165] Secher T, Normand S, Chamaillard M (2013). NOD2 prevents emergence of disease-predisposing microbiota. Gut Microbes.

[CR166] Seeliger S, Janssen PH, Schink B (2002). Energetics and kinetics of lactate fermentation to acetate and propionate via methylmalonyl-CoA or acrylyl-CoA. FEMS Microbiol Lett.

[CR167] Senkovich O, Cook WJ, Mirza S (2007). Structure of a complex of human lactoferrin N-lobe with pneumococcal surface protein a provides insight into microbial defense mechanism. J Mol Biol.

[CR168] Shadid R, Haarman M, Knol J (2007). Effects of galactooligosaccharide and long-chain fructooligosaccharide supplementation during pregnancy on maternal and neonatal microbiota and immunity–a randomized, double-blind, placebo-controlled study. Am J Clin Nutr.

[CR169] Singh NP, Singh UP, Singh B (2011). Activation of aryl hydrocarbon receptor (AhR) leads to reciprocal epigenetic regulation of FoxP3 and IL-17 expression and amelioration of experimental colitis. PLoS One.

[CR170] Singh N, Gurav A, Sivaprakasam S (2014). Activation of Gpr109a, receptor for niacin and the commensal metabolite butyrate, suppresses colonic inflammation and carcinogenesis. Immunity.

[CR171] Smits LP, Bouter KE, de Vos WM (2013). Therapeutic potential of fecal microbiota transplantation. Gastroenterology.

[CR172] Sokol H, Pigneur B, Watterlot L (2008). *Faecalibacterium prausnitzii* is an anti-inflammatory commensal bacterium identified by gut microbiota analysis of Crohn disease patients. Proc Natl Acad Sci USA.

[CR173] Sokol H, Seksik P, Furet JP (2009). Low counts of *Faecalibacterium prausnitzii* in colitis microbiota. Inflamm Bowel Dis.

[CR174] Sonnenberg GF, Artis D (2012). Innate lymphoid cell interactions with microbiota: implications for intestinal health and disease. Immunity.

[CR175] Sonnenberg GF, Fouser LA, Artis D (2011). Border patrol: regulation of immunity, inflammation and tissue homeostasis at barrier surfaces by IL-22. Nat Immunol.

[CR176] Southwick FS, Purich DL (1996). Intracellular pathogenesis of listeriosis. N Engl J Med.

[CR177] Spencer SP, Wilhelm C, Yang Q (2014). Adaptation of innate lymphoid cells to a micronutrient deficiency promotes type 2 barrier immunity. Science.

[CR178] Spits H, Cupedo T (2012). Innate lymphoid cells: emerging insights in development, lineage relationships, and function. Annu Rev Immunol.

[CR179] Spits H, Di Santo JP (2011). The expanding family of innate lymphoid cells: regulators and effectors of immunity and tissue remodeling. Nat Immunol.

[CR180] Stoppelenburg AJ, de Roock S, Hennus MP (2014). Elevated Th17 response in infants undergoing respiratory viral infection. Am J Pathol.

[CR181] Strauch UG, Obermeier F, Grunwald N (2005). Influence of intestinal bacteria on induction of regulatory T cells: lessons from a transfer model of colitis. Gut.

[CR182] Sun CM, Hall JA, Blank RB (2007). Small intestine lamina propria dendritic cells promote de novo generation of Foxp3 T reg cells via retinoic acid. J Exp Med.

[CR183] Takahashi H, Kanno T, Nakayamada S (2012). TGF-beta and retinoic acid induce the microRNA miR-10a, which targets Bcl-6 and constrains the plasticity of helper T cells. Nat Immunol.

[CR184] Tedelind S, Westberg F, Kjerrulf M (2007). Anti-inflammatory properties of the short-chain fatty acids acetate and propionate: a study with relevance to inflammatory bowel disease. World J Gastroenterol.

[CR185] Thurl S, Munzert M, Henker J (2010). Variation of human milk oligosaccharides in relation to milk groups and lactational periods. Br J Nutr.

[CR186] Treiner E, Duban L, Bahram S (2003). Selection of evolutionarily conserved mucosal-associated invariant T cells by MR1. Nature.

[CR187] van de Pavert SA, Ferreira M, Domingues RG (2014). Maternal retinoids control type 3 innate lymphoid cells and set the offspring immunity. Nature.

[CR188] van Liempt E, Bank CM, Mehta P (2006). Specificity of DC-SIGN for mannose- and fucose-containing glycans. FEBS Lett.

[CR189] van Neerven RJ (2014). The effects of milk and colostrum on allergy and infection: mechanisms and implications. Animal Front.

[CR190] van Neerven RJ, Knol EF, Heck JM (2012). Which factors in raw cow’s milk contribute to protection against allergies?. J Allergy Clin Immunol.

[CR191] van Nimwegen FA, Penders J, Stobberingh EE (2011). Mode and place of delivery, gastrointestinal microbiota, and their influence on asthma and atopy. J Allergy Clin Immunol.

[CR192] van Odijk J, Kull I, Borres MP (2003). Breastfeeding and allergic disease: a multidisciplinary review of the literature (1966–2001) on the mode of early feeding in infancy and its impact on later atopic manifestations. Allergy.

[CR193] Veldhoen M, Withers DR (2010). Immunology. Innate lymphoid cell relations. Science.

[CR194] Ventham NT, Kennedy NA, Nimmo ER (2013). Beyond gene discovery in inflammatory bowel disease: the emerging role of epigenetics. Gastroenterology.

[CR195] Verhasselt V (2010). Neonatal tolerance under breastfeeding influence: the presence of allergen and transforming growth factor-beta in breast milk protects the progeny from allergic asthma. J Pediatr.

[CR196] Vestman NR, Timby N, Holgerson PL (2013). Characterization and in vitro properties of oral lactobacilli in breastfed infants. BMC Microbiol.

[CR197] Vitali B, Cruciani F, Baldassarre ME (2012). Dietary supplementation with probiotics during late pregnancy: outcome on vaginal microbiota and cytokine secretion. BMC Microbiol.

[CR198] von Mutius E (2012). Maternal farm exposure/ingestion of unpasteurized cow’s milk and allergic disease. Curr Opin Gastroenterol.

[CR199] Vonarbourg C, Mortha A, Bui VL (2010). Regulated expression of nuclear receptor RORgammat confers distinct functional fates to NK cell receptor-expressing RORgammat(+) innate lymphocytes. Immunity.

[CR200] Vos AP, M’Rabet L, Stahl B (2007). Immune-modulatory effects and potential working mechanisms of orally applied nondigestible carbohydrates. Crit Rev Immunol.

[CR201] Wagner CL, Taylor SN, Johnson D (2008). Host factors in amniotic fluid and breast milk that contribute to gut maturation. Clin Rev Allergy Immunol.

[CR202] Watanabe Y, Nagai F, Morotomi M (2012). Characterization of Phascolarctobacterium succinatutens sp. nov., an asaccharolytic, succinate-utilizing bacterium isolated from human feces. Appl Environ Microbiol.

[CR203] Wolf HM, Fischer MB, Puhringer H (1994). Human serum IgA downregulates the release of inflammatory cytokines (tumor necrosis factor-alpha, interleukin-6) in human monocytes. Blood.

[CR204] Wolf HM, Hauber I, Gulle H (1996). Anti-inflammatory properties of human serum IgA: induction of IL-1 receptor antagonist and Fc alpha R (CD89)-mediated down-regulation of tumour necrosis factor-alpha (TNF-alpha) and IL-6 in human monocytes. Clin Exp Immunol.

[CR205] Wong AY, Chan EW, Chui CS (2014). The phenomenon of micronutrient deficiency among children in China: a systematic review of the literature. Public Health Nutr.

[CR206] Xu LB, Chen L, Gao W (2006). Bovine immune colostrum against 17 strains of diarrhea bacteria and in vitro and in vivo effects of its specific IgG. Vaccine.

[CR207] Zaph C, Du Y, Saenz SA (2008). Commensal-dependent expression of IL-25 regulates the IL-23-IL-17 axis in the intestine. J Exp Med.

[CR208] Zapolska-Downar D, Naruszewicz M (2009). Propionate reduces the cytokine-induced VCAM-1 and ICAM-1 expression by inhibiting nuclear factor-kappa B (NF-kappaB) activation. J Physiol Pharmacol.

[CR209] Zaura E, Nicu EA, Krom BP (2014). Acquiring and maintaining a normal oral microbiome: current perspective. Front Cell Infect Microbiol.

[CR210] Zhou L, Littman DR (2009). Transcriptional regulatory networks in Th17 cell differentiation. Curr Opin Immunol.

